# Clinical Outcomes of Bone‐Anchored Versus Socket‐Suspended Prostheses in Individuals With Transfemoral Amputation: A Systematic Review and Meta‐Analysis of Two‐Arm Comparative Studies

**DOI:** 10.1111/os.70369

**Published:** 2026-07-02

**Authors:** Janice Tan Sue Wei, Wen Xian Low, Chris Bretherton

**Affiliations:** ^1^ Barts and the London School of Medicine and Dentistry Queen Mary University of London London UK; ^2^ East Suffolk and North Essex NHS Foundation Trust London UK; ^3^ Bone and Joint Health, Blizard Institute Queen Mary University London London UK

**Keywords:** bone‐anchored prostheses, cost, gait, mobility, osseoperception, quality of life, range of motion, socket prostheses, transfemoral

## Abstract

Bone‐anchored prostheses (BAP) provide an alternative to traditional socket‐suspended prostheses (SSP) for individuals with transfemoral amputation (TFA) by addressing socket‐related complications. While most previous studies, primarily single‐arm trials, report positive outcomes with BAP, these findings may be overestimated due to lower preoperative baseline function among individuals using SSP prior to BAP implantation. This review aims to offer a more balanced comparison of functional and clinical outcomes between BAP and SSP by focusing exclusively on two‐arm comparative studies. A systematic review and meta‐analysis was conducted from December 2024 to May 2025, following PRISMA guidelines. Databases including PUBMED, EMBASE, Scopus, Cochrane, and Web of Science were searched. All studies meeting the inclusion criteria were cross‐sectional in design. Primary outcomes included mobility (Timed Up and Go [TUG]; Activities‐specific Balance Confidence [ABC]) and quality of life (Q‐TFA domains). Secondary outcomes: gait parameters, hip range of motion (ROM), osseoperception, and costs. Risk of bias was assessed using ROBINS‐I adapted for observational designs. Random‐effects meta‐analyses pooled mean differences (MD) or standardized mean differences (SMD) with 95% CIs. Twelve studies (BAP *n* = 200; SSP *n* = 244) met criteria. Pooled analyses showed no significant differences for TUG (MD 0.16 s, 95% CI −2.09 to 2.41; *I* ^2^ = 0%), ABC (MD 0.05, 95% CI −22.19 to 22.29; *I* ^2^ = 76.3), Q‐TFA Global (MD 1.14, 95% CI −22.02 to 24.30), Q‐TFA Prosthetic Mobility (MD 0.37, 95% CI −9.93 to 10.68), Q‐TFA Problems (MD –6.29, 95% CI –18.77 to 6.19), Q‐TFA Prosthesis Use (MD 1.18, 95% CI −2.36 to 4.71), and gait speed (MD −0.03 m/s, 95% CI −0.31 to 0.25). Hip ROM tended to be higher with BAP in two studies; osseoperception favored BAP in two studies; one study reported on lower long‐term cost with BAP. No comparative studies of infection were identified. While BAP is a promising alternative for individuals experiencing complications with SSP, its benefits for functional SSP users remain uncertain. The lack of comparative studies evaluating infection rates highlights an important area for future research.

## Graphical abstract

This systematic review and meta‐analysis compared bone‐anchored prostheses (BAP) and socket‐suspended prostheses (SSP) in individuals with transfemoral amputation (TFA) using only two‐arm comparative studies. Across 12 studies (BAP *n* = 200; SSP *n* = 244), no significant differences were found in mobility, quality of life, or gait. BAP showed possible advantages in hip ROM, osseoperception, and costs.

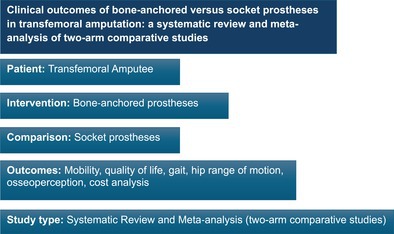

## 1 Introduction

It is estimated that there are 40 million amputees worldwide, with approximately 36 million (90%) having lower‐limb amputations [1]. About 26% of these are individuals with transfemoral amputation (TFA), which is associated with greater functional impairment due to the loss of the natural knee joint, resulting in increased biomechanical demands and reduced mobility [1]. This results in a significantly higher 1‐year mortality rate of 48%, compared to 29% for transtibial amputations, underscoring the need for greater attention [2]. Additionally, BAP have been more extensively studied in individuals with TFA, enabling more consistent evaluation and clinically meaningful comparison of outcomes.

The current treatment for lower limb amputation is a traditional socket‐suspended prostheses (SSP). However, many individuals undergoing lower‐extremity amputation experience complications associated with SSP, including skin ulcers, excessive perspiration, frequent need for refitting, and poor prosthetic fit due to residual limb volume fluctuation. Approximately 7% sustain fractures of the residual limb, and many report reduced confidence in mobility [3].

Therefore, bone‐anchored prostheses (BAP) were introduced through the process of osseointegration, which is defined as a direct structural and functional connection between living bone and the surface of a load‐bearing implant, enabling stable fixation without intervening soft tissue [4]. This enables direct force transfer to the bone, eliminating the need for a SSP and enhancing both comfort and functionality [5, 6]. Previous work has further characterized loading patterns at the bone–implant interface, demonstrating that forces are transmitted through the implant during daily activities, with distinct loading magnitudes and temporal profiles across functional tasks [7]. As a result, BAP are subjected to variable mechanical loads, with peak forces occurring during weight transfer and high‐demand activities [8]. While BAP improves functional outcomes, this increased mechanical loading may lead to localized bone resorption and compromise the integrity of the bone–implant interface [9]. Hence, the optimization of BAP outcomes depends on the ability to regulate mechanical loading within a safe physiological window [10].

The BAP are categorized into screw‐type and press‐fit implant systems. The Osseointegrated Prostheses for the Rehabilitation of Amputees (OPRA) is the first BAP developed and is the only screw‐type implant. It achieves fixation through a threaded titanium implant anchored directly into the bone, connected via an abutment and screw, with surface modifications designed to enhance stability and promote bone integration [11]. Subsequently, the press‐fit implant was developed and consists of various versions, including the Endo‐Exo Prosthesis (EEP), the Osseointegrated Prosthetics (OIP), and the Integral Leg Prosthesis (ILP), and the Osseointegrated Prosthetic Limb (OPL) [12].

However, BAP are associated with several drawbacks, including an increased risk of postoperative complications such as soft tissue infections, osteomyelitis, symptomatic neuroma, the need for revision surgery, and mechanical complications [13]. While most infections are superficial and can be managed conservatively, severe cases, such as septic failure, remain rare [14]. Additionally, BAP surgery involves strict selection criteria, with eligibility requiring patients to have complications with SSP, adequate bone quality, sufficient residual limb length, skeletal maturity, good overall health, and the ability to adhere to postoperative rehabilitation. Contraindications primarily relate to factors that increase infection risk or impair bone integration, including conditions such as active infection, vascular disease, diabetes, immunosuppression, smoking, and poor bone health [5]. BAP are also associated with a higher cost compared to SSP and is therefore recommended for SSP users facing challenges [15].

To date, many reviews showed promising results with BAP compared to SSP [16, 17, 18]. The latest review by Rehani et al. reported an overall positive patient experience with increased QoL, mobility, and prosthesis usage with BAP [19]. Although previous reviews have examined infection rates in BAP, none have directly compared infection rates between BAP and SSP [20, 21]. Another review also highlighted that while BAP showed consistent improvements in functional outcomes compared to SSP, the evidence remains limited due to methodological weaknesses, lack of standardized outcome measures, and inadequate control for confounding variables [22].

To date, a meta‐analysis of single‐arm studies by Tan et al. has reported significant improvements associated with BAP [23]. However, single‐arm studies compare outcomes in the same individuals before and after BAP surgery, typically when patients experience significant socket‐related complications. As a result, the preoperative baseline often reflects reduced function and quality of life, which can exaggerate the magnitude of observed improvements following intervention [23]. To address this limitation, the present review includes only comparative studies with two independent groups. These studies are not matched within subjects and do not rely on pre–post comparisons but instead evaluate differences between distinct cohorts. As such, it provides a complementary perspective to within‐subject designs when comparing outcomes between prosthetic approaches.

In this review, two‐arm comparative studies were defined as studies comparing two independent groups—individuals using BAP and those using SSP. Studies reporting outcomes at either a single time point (cross‐sectional) or across follow‐up periods were eligible for inclusion. The primary aim of this study was to compare clinical and functional outcomes between individuals using BAP and those using SSP. The null hypothesis (H_0_) was that there is no significant difference in mobility, quality of life, or gait outcomes between the two prosthetic approaches. The alternative hypothesis (H_1_) was that BAP provides superior clinical and functional outcomes compared with SSP. By focusing on two‐arm comparative studies, this review aims to provide a more representative comparison between BAP and SSP in established prosthesis users, rather than improvements observed following surgical conversion. These findings may help inform surgical decision‐making and support clinicians in setting realistic patient expectations when considering BAP.

## 2 Methods

### 2.1 Study Design

This systematic review aims to compare the clinical outcomes between BAP and SSP, following the Preferred Reporting Items for Systematic Reviews and Meta‐analyses (PRISMA) and Cochrane Handbook of Systematic Reviews of Interventions guidelines [24]. This review started in December 2024 and ended in May 2025. This review was registered on the International Prospective Register of Systematic Reviews (PROSPERO) under the registration number CRD420251137214.

### 2.2 Search Strategy

The search strategy was conducted following PICOS structure [25]. The following databases were used: PubMed, Cochrane, EMBASE, Web of Science and Scopus, with the last search carried out in 13/12/2024. Boolean operators (e.g., AND, OR, NOT) and relevant MeSH terms were used to facilitate a comprehensive search across databases. Search terms used are shown in Table 1. Outcomes were excluded to maximize the number of papers retrieved with no date limits. Reference lists and gray literature were manually screened, and authors of inaccessible papers were contacted via email.

**TABLE 1:** Search strategy.

Search	Query search terms	Number of results
(#1) Patient	Transfemoral OR “above knee” OR “lower limb” OR “lower extremity” OR “transfemoral amput*” OR “lower amputation”	111,809
(#2) Intervention	“Bone‐anchored” OR “bone anchored” OR osseointegrat* OR “leg prosthe*” OR “leg‐prosthe*” OR “press‐fit” OR “press fit” OR “screw‐type” OR “screw type” OR OPRA OR EEP OR OIP OR ILP OR OPL OR Bone‐anchored prosthesis[MeSH] OR Osseointegration[MeSH] OR Prosthesis Implantation/methods[MeSH]	84,487
(#3) Comparison	Socket OR “socket‐suspension” OR “socket‐suspended”	63,400
Total	#1 AND #2 AND #3	159

Abbreviations: EEP, Endo‐Exo Prosthesis; ILP, Integral Leg Prosthesis; OIP, Osseointegrated Prosthetics; OPL, Osseointegrated Prosthetic Limb; OPRA, Osseointegrated Prostheses for the Rehabilitation of Amputees.

### 2.3 PICOS Description

The PICOS (Population, Intervention, Comparator, Outcome, Study design) framework was used in conducting this review, shown in Table 2. The target population group is TFA, including both unilateral and bilateral. The intervention included any type of BAP, such as OPRA, OIP, TOPS, and ILP systems. The comparator group consisted exclusively of individuals using SSP.

**TABLE 2:** Inclusion/exclusion criteria.

	Inclusion	Exclusion
Population	Human subjects Unilateral or bilateral TFA Over 18 years old	Amputation at other level
Intervention	BAP users	Other form of prostheses Not direct skeletal fixation, not robotic, microprocessor knee
Comparators	SSP users	Non‐SSP users
Outcomes	Quality of life, mobility, gait analysis, hip range of motion, osseoperception, cost analysis, infection	
Study design	Two‐arm comparative studies only (cross‐sectional, prospective cohort, retrospective) RCTs or NRCTs	Other languages other than English Single‐arm trial Reviews, case series, case reports and editorial pieces Animal studies Abstracts, commentaries and expert opinion studies

Abbreviations: BAP, bone‐anchored prostheses; NRCTs, non‐randomized controlled trials; RCTs, randomized controlled trials; SSP, socket‐suspended prostheses; TFA, individuals with transfemoral amputation.

The outcomes included quality of life, mobility, gait analysis, hip range of motion, osseoperception, cost analysis, and infection. Only two‐arm comparative studies were included. These were defined as studies comparing two independent groups, namely individuals using BAP (intervention group) and those using SSP (comparator group), either at a single time point (cross‐sectional) or across follow‐up periods. Single‐arm studies based on within‐subject comparisons, where participants serve as their own control, were excluded.

### 2.4 Study Selection

The two authors (JT and WX) independently reviewed the retrieved studies to determine their eligibility. Firstly, the titles and abstracts of the studies were reviewed, followed by further assessment of the full texts for relevant studies. Disagreements were resolved through discussion, consulting a third author if necessary for a final decision. The review authors documented all reasons for exclusion by labelling them on the Rayyan website [26].

### 2.5 Data Extraction

Data was collected by the primary author and checked by the secondary author, with any discrepancies resolved by discussion. Data extracted were synthesized using Excel and Word document. The following data were extracted: author, year of publication, country, number of participants, type of prostheses, age of implantation, BMI, reason for amputation, time since amputation, and time with BAP.

### 2.6 Methodological Quality

The ROBINS‐I V2 tool was used to evaluate the risk of bias in non‐randomized controlled trials (NRCTs) across seven domains: confounding variables, participant selection, intervention classification, deviations from intended interventions, missing data, outcome measurement, and selection of reported results [27].

The GRADEpro tool (Grading of Recommendations Assessment, Development and Evaluation certainty of evidence) was used to assess the level of evidence of included studies [28]. This is shown in Appendix S1.

### 2.7 Statistical Methods

A meta‐analysis will be attempted where three or more studies report the same outcome measures and provide mean and standard deviation data. This is performed by calculating the mean difference with an inverse variance random‐effects model with a 95% confidence interval. Comprehensive data extraction and statistical outputs are presented in Appendix S2.

If there are less than three studies per outcome measurement, a best‐evidence synthesis of outcomes was performed, presented in the form of a narrative summary or a tabular format.

### 2.8 Ethics

Ethical approval is not required for this review as no patients are involved.

## 3 Results

### 3.1 Description of Studies

**FIGURE 1:**
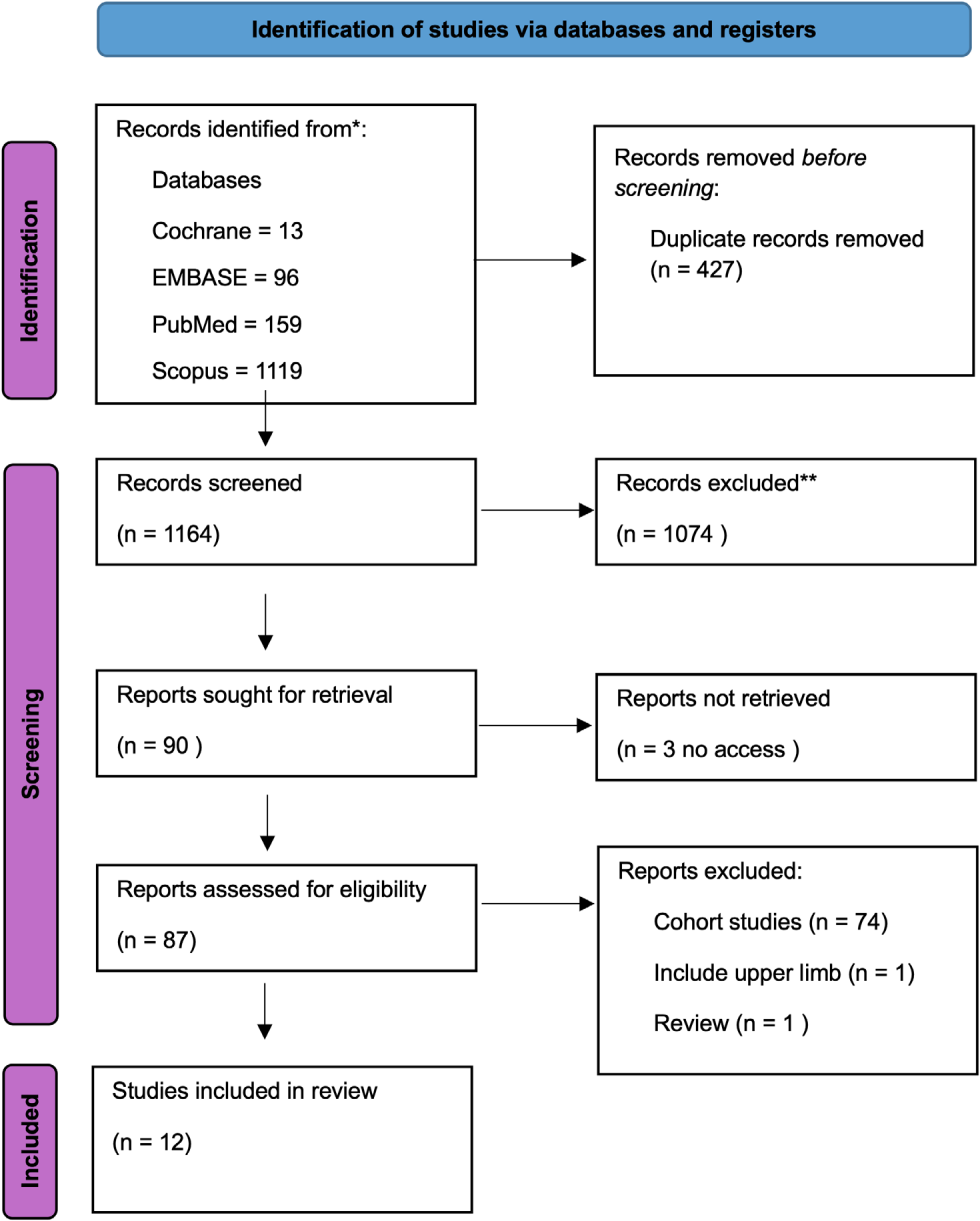
PRISMA chart flow diagram.

**FIGURE 2:**
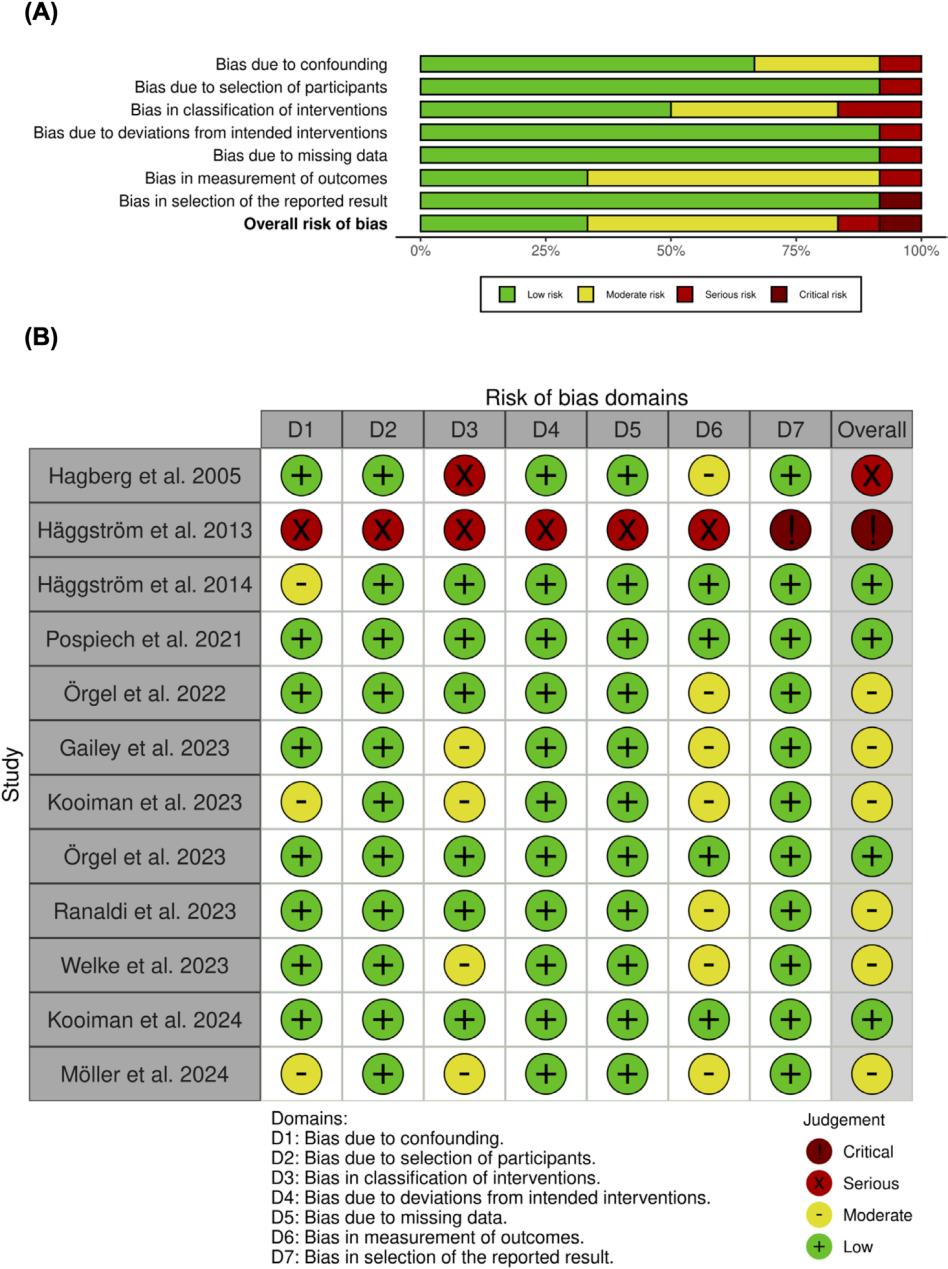
The ROBINS‐I V2 risk of bias assessment of included studies. (A) Risk of bias graph for included studies; (B) risk of bias summary for included studies.

The screening process identified 12 cross‐sectional studies [29, 30, 31, 32, 33, 34, 35, 36, 37, 38, 39, 40] based on inclusion and exclusion criteria, illustrated in Figure 1 [29, 30, 31, 32, 33, 34, 35, 36, 37, 38, 39, 40]. The risk of bias assessment with ROBINS‐I V2 is shown in Figure 2.

Study characteristics are summarized in Tables 3a, 3b, 3c, 3d, 3e, 3f–3a, 3b, 3c, 3d, 3e, 3f and stratified by outcome: Table 3a (mobility), Table 3b (quality of life), Table 3c (gait analysis), Table 3d (hip range of motion), Table 3e (osseoperception), and Table 3f (cost analysis). This review comprised 244 participants with SSP and 200 participants with BAP. It should be noted that as some studies originated from the same institutions or research groups, there is a possibility of overlapping participant cohorts.

**TABLE 3a:** Summary of study characteristics: mobility.

Study (year)	Country	Participants		Type of BAP	Outcome measurements					
					TUG	6MWT	2MWT	ABC	PLUS‐M	PMQ 2.0
		SSP	BAP							
Orgel et al. (2022) [36]	Germany	36	33	TOPS	NR	NR	NR	NR	NR	BAP group significantly better, *p* < 0.000
Gailey et al. (2023) [34]	USA	11	11	ILP	NR	NR	NR	*p* = 0.05	*p* = 0.05	NR
Kooiman et al. (2023) [35]	The Netherlands	10	10	NR (*Press‐fit*)^a^	NR	NR	BAP group: 155 ± 22 m*	BAP group: 90 ± 9*	NR	NR
							SSP group: 162 ± 29 m*	SSP group: 90 ± 11*		
Welke et al. (2023) [31]	Germany	17	20	TOPS	*p* = 0.876	*p* = 0.876	NR	NR	NR	NR
Orgel et al. (2023) [33]	Germany	25	25	EEP (TOPS)^b^	NR	NR	NR	NR	NR	BAP group significantly better, *p* < 0.001
Moller et al. (2024) [29]	Sweden	8	8	OPRA	*p* = 0.094	*p* = 0.094	NR	NR	*p* = 0.161	NR

Abbreviations: 2MWT, 2‐Minute Walk Test; 6MWT, 6‐Minute Walk Test; ABC, Activities‐specific Balance Confidence; BAP, bone‐anchored prostheses; ILP, Integral Leg Prosthesis; NR, not reported; OPRA, Osseointegrated Prostheses for the Rehabilitation of Amputees; PLUS‐M, Prosthetic Limb Users Survey of Mobility; PMQ 2.0, Prosthesis Mobility Questionnaire 2.0; SSP, socket‐suspended prostheses; TOPS, Transcutaneous Osseointegrated Prosthetic System; TUG, Timed Up and Go Test.

^a^ Type of BAP not stated, but press‐fit design reported.

^b^ The EEP is a type of TOPS.

* *p* value not reported.

**TABLE 3b:** Summary of study characteristics: quality of life.

Study (year)	Country	Participants		Type of BAP	Outcome measurements			
					Q‐TFA (global, mobility, problem, prosthetic use)	EQ‐5D‐3L	SAT‐PRO	SF‐36
		SSP	BAP					
Pospiech et al. (2021) [37]	Germany	22	17	OIP	Global: BAP group significantly better, *p* = 0.022 Mobility: *p* = 0.051 Problem: BAP group significantly better, *p* < 0.001 Prosthetic use: *p* = 0.146	TTO‐VS: *p* = 0.723 VAS‐VS: *p* = 0.497	NR	NR
Orgel et al. (2022) [36]	Germany	36	33	TOPS	*p* = 0.000	BAP group significantly better, *p* = 0.004	BAP group significantly better, *p* = 0.000	NR
Kooiman et al. (2023) [35]	The Netherlands	10	10	NR (*Press‐fit*)^a^	Global: *p* = 0.529 Mobility: *p* = 1 Problem: *p* = 0.481 Prosthetic use: *p* = 0.481	NR	NR	*p* = 0.876
Welke et al. (2023) [31]	Germany	17	20	TOPS	Global: *p* = 0.354 Mobility: *p* = 0.493 Problem: *p* = 0.063 Prosthetic use: *p* = 0.745	NR	NR	MCS: *p* = 0.293 PCS: *p* = 0.892
Moller et al. (2024) [29]	Sweden	8	8	OPRA	Prosthesis use: *p* = 0.89	NR	NR	NR

Abbreviations: BAP, bone‐anchored prostheses; EEP, Endo‐Exo Prosthesis; EQ‐5D‐3L; European Questionnaire 5‐Dimension, 3‐Level; MCS, Mental Component Summary; NR, not reported; OIP, Osseointegrated Prosthetics; OPRA, Osseointegrated Prostheses for the Rehabilitation of Amputees; PCS, Physical Component Summary; Q‐TFA, Questionnaire for Persons with a Transfemoral Amputation; SAT‐PRO, Satisfaction with Prosthesis Questionnaire; SF‐36, 36‐Item Short Form Health Survey; SSP, socket‐suspended prostheses; TOPS, Transcutaneous Osseointegrated Prosthetic System; TTO‐VS, time trade‐off value set; VAS‐VS, visual analog scale value set.

^a^ Type of BAP not stated, but press‐fit design reported.

**TABLE 3c:** Summary of study characteristics: gait analysis.

Study (year)	Country	Participants		Type of BAP	Outcome measurements				
					Single support time	Step length	Step time	Step width	Gait speed
		SSP	BAP						
Gailey et al. (2023) [34]	USA	11	11	ILP	*p* = 0.95	NR	NR	NR	*p* = 0.07
Ranaldi et al. (2023) [32]	Italy, Sweden	9	9	OPRA	NR	*p* = 0.693	NR	BAP group significantly lower, *p* = 0.001	*p* = 0.438
Welke et al. (2023) [31]	Germany	17	20	TOPS	*p* = 0.387	*p* = 0.574	*p* = 0.882	BAP group significantly lower, *p* = 0.001	*p* = 0.843
Kooiman et al. (2024) [30]	The Netherlands	10	10	NR	No significant differences*	No significant differences*	No significant differences*	No significant differences*	No significant differences*

Abbreviations: BAP, bone‐anchored prostheses; ILP, Integral Leg Prosthesis; NR, not reported; OPRA, Osseointegrated Prostheses for the Rehabilitation of Amputees; SSP, socket‐suspended prostheses; TOPS, Transcutaneous Osseointegrated Prosthetic System.

* *p* value not reported.

**TABLE 3d:** Summary of study characteristics: hip range of motion.

Study (year)	Country	Participants		Type of BAP	Outcome measurements		
					Flexion‐extension	Abduction–adduction	Rotation
		SSP	BAP				
Hagberg et al. (2005) [40]	Sweden, UK	43	20	OPRA^a^	BAP group: 127° ± 12* SSP group: 97° ± 13.5*	BAP group: 48° ± 5.9* SSP group: 45° ± 8.3*	BAP group: 48° ± 9.5 SSP group: 0° ± 0.8 BAP group significantly higher, *p* < 0.001
Welke et al. (2023) [31]	Germany	17	20	TOPS	BAP group: 48.2° ± 6.2 SSP group: 44.3° ± 7.4 No significant difference: *p* = 0.064	BAP group: 23.2° ± 8.2 SSP group: 18° ± 5.5 BAP group significantly higher, *p* = 0.03	BAP group: 10.8° ± 3.1 SSP group: 10.2° ± 3.6 No significant difference: *p* = 0.403

Abbreviations: BAP, bone‐anchored prostheses; OPRA, Osseointegrated Prostheses for the Rehabilitation of Amputees; SSP, socket‐suspended prostheses; TOPS, Transcutaneous Osseointegrated Prosthetic System.

^a^ OPRA not explicitly stated, but implied due to its osseointegration method with a titanium fixture and its Swedish origin.

* *p* value not reported.

**TABLE 3e:** Summary of study characteristics: osseoperception.

Study (year)	Country	Participants		Type of BAP	Outcome measurements	
					Detection thresholds of vibrometric stimuli (8, 16, 32, 64, 125, and 250 Hz)	Identify shore hardness of different materials (V1, V2, V3)
		SSP	BAP			
Haggstrom et al. (2014) [38]	Sweden	17	17	OPRA	BAP group showed better ability to detect high frequency vibrations (125 and 250 Hz) than the SSP group (*p* = 0.01 and 0.03) respectively	NR
Orgel et al. (2023) [33]	Germany	25	25	EEP (TOPS)^a^	NR	Material V3 showed no significant difference (*p* = 0.158). Material V1 and V2 had significantly higher scores in the BAP group (*p* < 0.001 for both)

Abbreviations: BAP, bone‐anchored prostheses; EEP, Endo‐Exo Prosthesis; OPRA, Osseointegrated Prostheses for the Rehabilitation of Amputees; SSP, Socket‐Suspended Prostheses; TOPS, Transcutaneous Osseointegrated Prosthetic System.

^a^ The EEP is a type of TOPS.

**TABLE 3f:** Summary of study characteristics: cost analysis.

Study (year)	Country	Participants		Type of BAP	Outcome measurements		
					Prosthetic cost (annual mean cost of new prostheses, service, repair, and adjustment)	Manufacturing cost	Number of annual visit
		SSP	BAP				
Haggstrom et al. (2013) [39]	Sweden	36	20	OPRA	BAP group: €3149 SSP group: €3672 No significant difference: *p* = 0.632	BAP group: €9370 ± 6441* SSP group: €4890 ± 1758*	BAP group: 3.1 SSP group: 7.2 BAP group significantly lower, *p* < 0.0001

Abbreviations: BAP, bone‐anchored prostheses; OPRA, Osseointegrated Prostheses for the Rehabilitation of Amputees; SSP, socket‐suspended prostheses.

* *p* value not reported.

Five studies took place in Sweden [29, 32, 38, 39, 40], four in Germany [31, 33, 36, 37], two in the Netherlands [30, 35], one in UK [40], one in Italy [32] and one in USA [34]. Six studies reported on mobility [29, 31, 33, 34, 35, 36], five on quality of life [29, 31, 35, 36, 37] four on gait analysis [30, 31, 32, 34], two on hip range of motion [31, 40], two on osseoperception [33, 38], and one on cost analysis [39]. No studies on complications or infections met the inclusion criteria. Regarding the types of BAPs, five studies used OPRA [29, 32, 38, 39, 40], three used TOPS [31, 33, 36], one used ILP [34], and one used OIP [37].

The characteristics of the patients evaluated are reported in Table 4. The SSP group included 161 males and 76 females, while the BAP group comprised 123 males and 79 females. It should be noted that one study by Gailey et al. did not report the gender of participants [34]. Participants' ages ranged from 43.2 to 62 years for the SSP group and 44.6 to 59 years for the BAP group. Trauma was the most common reason for amputation in both groups. The body mass index (BMI) ranged from 25.7 to 31.2 for the SSP group and 25.14 to 31.7 for the BAP group. The time since amputation varied between 149.7 months to 29 years in the SSP group and 86.7 months to 25 years in the BAP group. The time with BAP ranged from 2 to 16 years.

**TABLE 4:** Patient demographics.

Study	SSP					BAP					
	Number of participants, n (M:F)	Age, years (mean ± SD/median and range)	BMI (mean ± SD)	Reason for amputation, n	Time since amputation (mean ± SD/median and range)	Number of participants, n (M:F)	Age, years (mean ± SD/median and range)	BMI (mean ± SD)	Reason for amputation, n	Time since amputation (mean ± SD/median and range)	Time with BAP (mean ± SD/median and range)
Hagberg et al. (2005) [40]	43 (32:11)	51 ± 11.7	27.5 ± 4	Trauma [30], Tumor [11], Other [2]	29 ± 14.7 years	20 (15:5)	46 ± 11.3	28.6 ± 4.6	Trauma [14], Tumor [4], Other [2]	19 ± 11.3 years	5 (3–10)^a^ years
Haggstrom et al. (2013) [39]	36 (30:6)	51.3 ± 9.3	NR	Trauma [27], Tumor [7], Other [2]	25.7 ± 13.1 years	20 (11:9)	56.6 ± 11.7	NR	Trauma [13], Tumor [5], Other [2]	23.2 ± 13.5 years	9.8 ± 3.5 years
Haggstrom et al. (2014) [38]	17 (11:6)	43.2 (29–63)^a^	NR	Trauma [11], Tumor [6]	18 (2–34)* years	17 (8:9)	44.6 (23–63)^a^	NR	Trauma [11], Tumor [6]	14.5 (2–42)^a^ years	2 years
Pospiech et al. (2021) [37]	17 (12:5)	47 ± 12.3	25.8 ± 3.3	Trauma [12], Tumor [4], Other [1]	241 ± 17 months	22 (17:5)	48.7 ± 8.3	27.1 ± 4.5	Trauma [15], Tumor [3], Other [4]	230 ± 138 months	66.8 ± 2 42.4 months
Orgel et al. (2022) [36]	36 (18:18)	48.6 ± 13	26.9 ± 5.2	Trauma [23], Tumor [3], Vascular [4], Sepsis [2], Iatrogenic [3], Missing data [1]	NR	33 (17:16)	52.1 ± 9.7	29.5 ± 6.5	Trauma [21], Tumor [3], Vascular [4], Sepsis [1], Iatrogenic [4]	NR	30.5 ± 41.5 months
Gailey et al. (2023) [34]	11	49.6 ± 16	31.2 ± 5	NR	149.7 ± 193.8 months	11	44.7 ± 14.9	31.7 ± 6.9	NR	86.7 ± 102.3 months	NR
Kooiman et al. (2023) [35]	10 (5:5)	56 ± 13	NR	Trauma [8], Cancer [1], Infection [1]	27 ± 14 years	10 (6:4)	59 ± 15	NR	Trauma [5], Cancer [4], Congenital [1]	25 ± 17 years	5 ± 2 years
Orgel et al. (2023) [33]	25 (19:6)	52.3 ± 13.1	27.8 ± 5.3	Trauma [19], Vascular [3], Tumor [3]	16.7 ± 11.3 years	25 (15:10)	50.6 ± 9.4	26.5 ± 3.6	Trauma [17], Vascular [6], Tumor [2]	15.4 ± 11.6 years	NR
Ranaldi et al. (2023) [32]	18 (9:9)	59 ± 9	26.73 ± 5.56	NR	NR	17 (9:8)	51 ± 13	27.55 ± 5.45	NR	NR	NR
Welke et al. (2023) [31]	17 (13:4)	62 ± 14.6	25.7 ± 4	Trauma [9], Tumor [4], PAD [3], Other [1]	25.2 ± 18.7 years	20 (12:8)	54 ± 8.2	28.2 ± 4.5	Trauma [12], Tumor [4], PAD [2], Sepsis [1], Other [1]	23.7 ± 13.2 years	6.6 ± 2.4 years
Kooiman et al. (2024) [30]	10 (5:5)	56 ± 13	25.83 ± 6.78	Trauma [8], Cancer [1], Infection [1]	27 ± 14 years	10 (6:4)	59 ± 15	25.14 ± 4.71	Trauma [5], Cancer [4], Congenital [1]	25 ± 17 years	5 ± 2 years
Moller et al. (2024) [29]	8 (7:1)	46 ± 9.5	NR	Tumor [6], Trauma [2]	23 ± 18.5 years	8 (7:1)	52 ± 15.3	NR	Trauma [6], Tumor [2]	16 ± 11.2 years	4–16 years

Abbreviations: BAP, bone‐anchored prostheses; BMI, body mass index; F, Female; M, Male; *n*, number of participants; NR, not reported; SD, standard deviation; SSP, Socket‐Suspended Prostheses.

^a^ Median and range provided instead, as mean ± SD is unavailable.

### 3.2 Mobility

Mobility was assessed in six studies [29, 31, 33, 34, 35, 36]. For Performance‐Based Outcome Measures (PBOMs), three studies reported on the Timed Up and Go (TUG) test [31, 34, 35], two on the 6‐Minute Walk Test (6MWT) [29, 31], and one on the 2‐Minute Walk Test (2MWT) [35]. For Patient‐Reported Outcome Measures (PROMs), three studies reported on the Activities‐specific Balance Confidence (ABC) scale [29, 34, 35], two on the Prosthetic Limb Users Survey of Mobility (PLUS‐M) [29, 34], two on the Prosthesis Mobility Questionnaire 2.0 (PMQ 2.0) [33, 36]. The ABC scale was used to assess prosthesis users' perceived balance confidence in performing daily tasks. The PLUS‐M was used to evaluate prosthesis users' perceived ability to ambulate with their prosthesis. Findings are presented in Table 3a.

#### 3.2.1 Meta Analysis for Mobility

**FIGURE 3:**
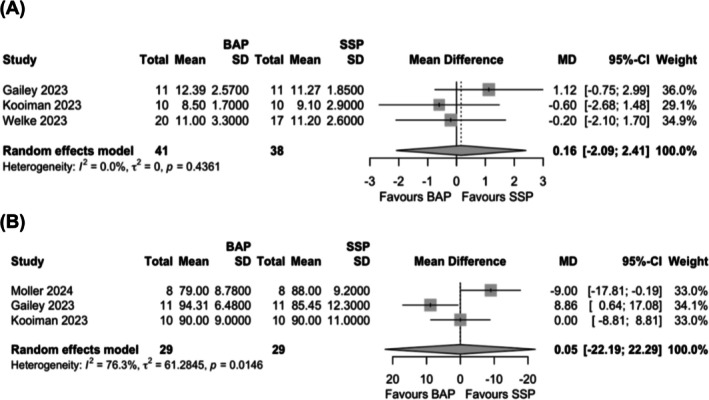
Forest plot comparing BAP versus SSP. (A) Timed Up and Go (TUG) test; (B) Activities‐specific Balance Confidence (ABC) scale.

A meta‐analysis was conducted for both TUG and ABC outcomes. The TUG was evaluated across three studies, yielding a mean difference of 0.16 (95% CI: −2.09 to 2.41; *p* = 0.789) (Figure 3A) [31, 34, 35]. Similarly, the ABC was assessed in three studies, showing a mean difference of 0.05 (95% CI: −22.19 to 22.29; *p* = 0.993) (Figure 3B) [29, 34, 35]. Overall, results for mobility showed no significant difference between BAP and SSP.

### 3.3 Quality of Life

Quality of life (QoL) was assessed in five studies, with five reporting on the Questionnaire for Persons with a Transfemoral Amputation (Q‐TFA) [29, 31, 35, 36, 37], two on the European Questionnaire 5‐Dimension (EQ‐5D), one on the 36‐Item Short Form Survey (SF‐36), and one on the Satisfaction with Prosthesis Questionnaire (SAT‐PRO). Findings are presented in Table 3b.

#### 3.3.1 Meta Analysis for Quality of Life

**FIGURE 4:**
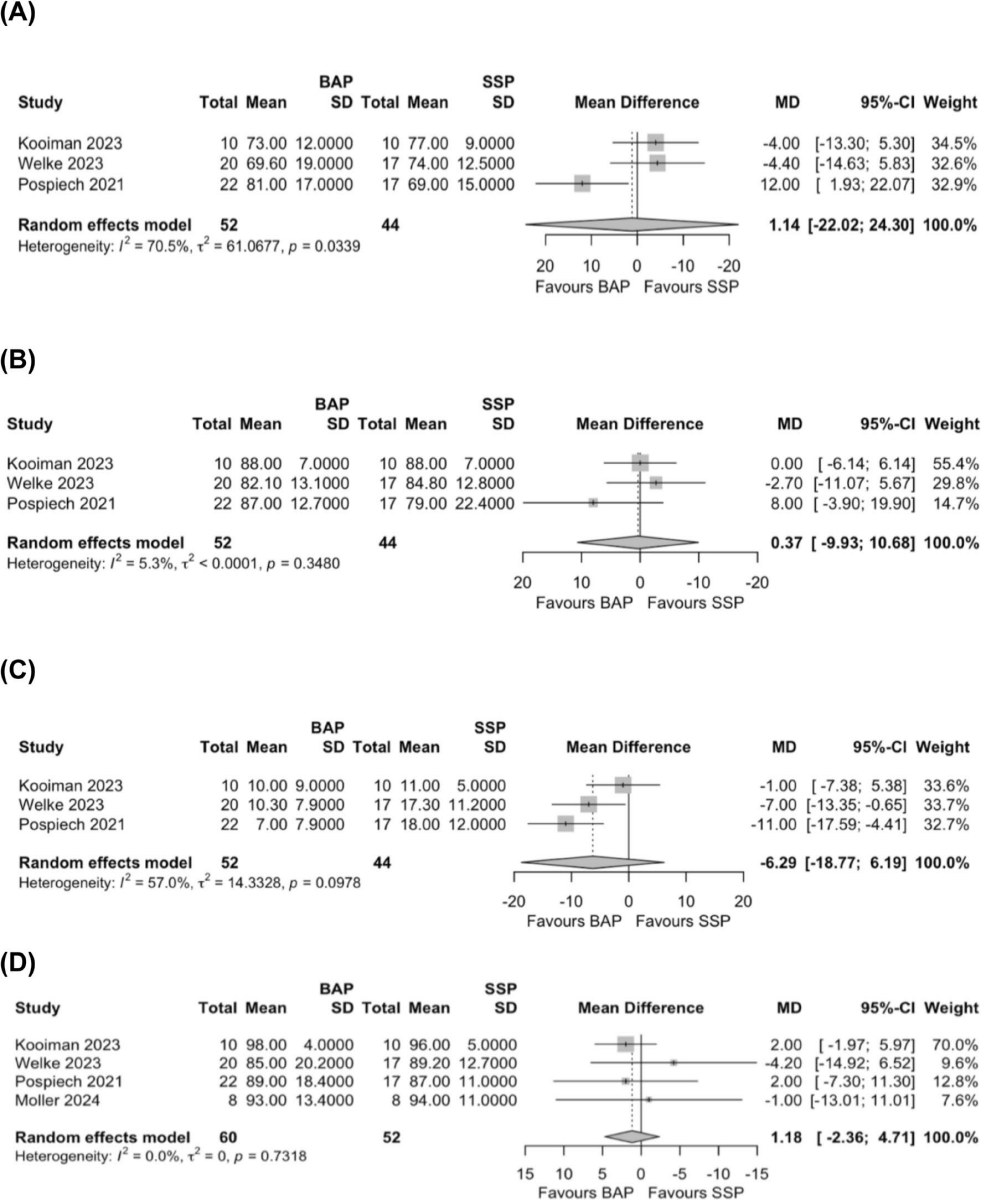
Forest plot for Questionnaire for Persons with a Transfemoral Amputation (Q‐TFA) comparing BAP versus SSP. (A) Global health; (B) Prosthetic mobility; (C) Problem; (D) Prosthesis use.

Meta‐Analysis Was Conducted for the Four Domains of the Q‐TFA: Global Health, Prosthetic Mobility, Problems, and Prosthesis Use. Global Health Was Assessed in Three Studies, Yielding a Mean Difference of 1.14 (95% CI: −22.02 to 24.30; *p* = 0.852) (Figure 4A) [31, 35, 37]. Prosthetic Mobility Was Assessed in Three Studies, With a Mean Difference of 0.37 (95% CI: −9.93 to 10.68; *p* = 0.890) (Figure 4B) [31, 35, 37]. The problems domain was assessed in three studies, showing a mean difference of −6.29 (95% CI: −18.77 to 6.19; *p* = 0.162) (Figure 4C) [31, 35, 37]. Prosthesis use was assessed in four studies, showing a mean difference of 1.18 (95% CI: −2.36 to 4.71; *p* = 0.368) (Figure 4D) [29, 31, 35, 37].

### 3.4 Gait Analysis

Studies have reported various outcome measurements for gait analysis. This review focuses on the most widely reported spatiotemporal parameters, including single‐support time, step length, step time, step width, and gait speed, as identified in four studies. This is reported in four studies [30, 31, 32, 34]. Findings are presented in Table 3c.

Two studies reported on single‐support time [31, 34], three on step length [30, 31, 32], two on step time [30, 31], and four on gait speed [30, 31, 32, 34]. All studies found no significant difference between the BAP group and the SSP group. In contrast, for step width, two studies reported a significantly lower step width in the BAP group (*p* = 0.001) [31, 32], while one study found no significant difference [30].

#### 3.4.1 Meta Analysis for Gait

**FIGURE 5:**
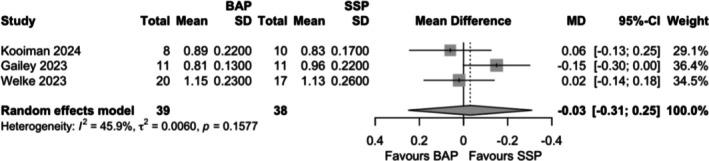
Forest plot for gait speed comparing BAP versus SSP.

Meta‐analysis was conducted for the gait speed. This was assessed in three studies, showing a mean difference of −0.03 (95% CI: −0.31 to 0.25; *p* = 0.687) (Figure 5) [30, 31, 34].

### 3.5 Hip Range of Motion

Hip range of motion (ROM) was reported in two studies, assessing flexion‐extension, abduction‐adduction, and hip rotation [31, 40]. Findings are presented in Table 3d. For flexion‐extension ROM, Hagberg et al. reported increase ROM in the BAP group (127° ± 12) than the SSP group (97° ± 13.5) [40]. Similarly, Welke et al. found higher ROM in the BAP group (48.2° ± 6.2) compared to the SSP group (44.3° ± 7.4), though this difference was not statistically significant (*p* = 0.064) [31]. For abduction‐adduction ROM, Hagberg et al. observed slightly higher ROM in the BAP group (48° ± 5.9) than the SSP group (45° ± 8.3) [40]. In contrast, Welke et al. reported a significantly greater ROM in the BAP group (23.2° ± 8.2) compared to the SSP group (18° ± 5.5, *p* = 0.03) [31]. When comparing hip rotation ROM between both groups, Hagberg et al. showed significantly greater ROM in the BAP group compared to the SSP group (*p* < 0.001) [40]. In contrast, Welke et al. found no significant difference (*p* = 0.403) [31].

### 3.6 Osseoperception

Osseoperception is evaluated in two studies [33, 38]. Findings are presented in Table 3e. Haggstrom et al. measured by this by measuring detection threshold of vibrotactile stimulation in SSP and BAP in frequencies: 8, 16, 32, 64, 125, and 250 Hz [38]. The results showed that the BAP group had better ability to detect high frequency vibrations (125 and 250 Hz) than the SSP group (*p* = 0.01 and 0.03, respectively). There was no significant different between groups at the lower frequencies [38].

Orgel et al. assessed osseoperception through three concealed tests (V1, V2, V3), where SSP and BAP group participants ranked materials by shore hardness [33]. V3 showed no significant difference between the groups (*p* = 0.158), whereas V1 and V2 had significantly higher scores in the BAP group compared to the SSP group (*p* < 0.001 for both). Overall, the BAP group scored significantly higher across V1–V3 (7.1 ± 1.1) compared to the SSP group (5.4 ± 1.2, *p* < 0.001) [33].

### 3.7 Cost Analysis

Only Haggstrom et al. compared prosthetic cost between SSP and BAP group. Findings are presented in Table 3f. Results showed that the BAP group had 14% lower mean total annual cost of new prostheses, services, repairs and adjustments, compared to SSP group [39] (SSP €3672, BAP €3149, *p* = 0.632). The average manufacturing cost was €4890 ± €1758 for SSP prostheses and €9370 ± €6441 for BAP. This was accompanied by significantly more annual visits in the SSP group (7.2 visits) compared to the BAP group (3.1 visits, *p* < 0.0001) [39].

### 3.8 Summary of Findings

The summary of findings table (Table 5) presents the main outcomes included in the meta‐analysis and reports the GRADE assessment of the certainty of the evidence.

**TABLE 5:** Summary of findings.

Outcome	Number of studies	Number of participants (BAP:SSP)	Absolute effect (95% CI)	Certainty of evidence (GRADE)	Comment
TUG	3	41:38	MD 0.16 higher (2.09 lower to 2.41 higher)	⨁◯◯◯ Very low^a^, ^b^, ^c^, ^d^	Results showed no significant difference. The evidence is based on a small number of studies with limited sample size, all NRCTs carrying a moderate risk of bias.
ABC	3	29:29	MD 0.05 higher (22.19 lower to 22.29 higher)	⨁◯◯◯ Very low^a^, ^b^, ^c^, ^d^	Results showed no significant difference. The evidence is based on a small number of studies with limited sample size, all NRCTs carrying a moderate risk of bias.
Q‐TFA Global	3	52:44	MD 1.14 higher (22.02 lower to 24.3 higher)	⨁◯◯◯ Very low^a^, ^b^, ^c^, ^d^, ^e^, ^f^	Results showed no significant difference. The evidence is limited by the small number of studies and sample size, all NRCTs with moderate risk of bias. Considerable heterogeneity was also present, further reducing certainty.
Q‐TFA Prosthetic Mobility	3	52:44	MD 0.37 higher (9.93 lower to 10.68 higher)	⨁⨁◯◯ Low^a^, ^b^, ^c^, ^d^, ^f^	Results showed no significant difference. The evidence is based on a small number of studies with limited sample size, all NRCTs carrying a moderate risk of bias.
Q‐TFA Problem	3	52:44	MD 6.29 lower (18.77 lower to 6.19 higher)	⨁◯◯◯ Very low^a^, ^b^, ^c^, ^d^, ^e^, ^f^	Results showed no significant difference. The evidence is limited by the small number of studies and sample size, all NRCTs with moderate risk of bias. Considerable heterogeneity was also present, further reducing certainty.
Q‐TFA Prosthetic Use	4	60:52	MD 1.18 higher (2.36 lower to 4.71 higher)	⨁◯◯◯ Very low^a^, ^b^, ^c^, ^f^	Results showed no significant difference, with all included NRCTs carrying a moderate risk of bias.
Gait speed	3	39:38	MD 0.03 lower (0.31 lower to 0.25 higher)	⨁◯◯◯ Very low^a^, ^b^, ^c^, ^d^	Results showed no significant difference. The evidence is based on a small number of studies with limited sample size, all NRCTs carrying a moderate risk of bias.

*Note: Patient:* Individuals with transfemoral amputations (TFA). *Intervention:* Bone‐anchored prostheses (BAP). *Comparison:* Socket‐suspended prostheses (SSP). *Outcomes:* Mobility, quality of life, gait, hip range of motion, osseoperception, cost analysis. *Study type:* Systematic review and meta‐analysis (two‐arm comparative studies).

Abbreviations: ABC, Activities‐specific Balance Confidence; BAP, bone‐anchored prostheses; CI, confidence interval; GRADE, grading of recommendations assessment, development and evaluation certainty of evidence; MD, mean difference; NRCTs, non‐randomized controlled trials; Q‐TFA, questionnaire for a persons with a transfemoral amputation; SSP, socket‐suspended prostheses; TFA, individuals with transfemoral amputation; TUG, Timed Up and Go Test.

^a^ Bias due to confounding.

^b^ Bias in classification of intervention.

^c^ Bias in measurement of outcome.

^d^ Less than three studies included, giving small sample size.

^e^ High heterogeneity (*I* ^2^ > 50%) in forest plot.

^f^ One study was not included in the meta‐analysis as no raw data were provided but reported a significant result.

## 4 Discussion

To date, this is the first systematic review that exclusively includes cross‐sectional studies. The BAP group generally showed improved hip ROM, osseoperception, and lower long‐term costs. However, mobility assessments, gait, and Q‐TFA showed no significant differences.

### 4.1 Mobility

Studies reporting Performance‐Based Outcome Measures (PBOMs) such as 6MWT and TUG, showed no significant differences between the BAP and the SSP group [29, 30, 31, 34]. This contrasts with previous single‐arm trials that reported significant improvements in 6MWT and TUG scores when comparing the pre‐operative state of SSP users to their post‐operative state with BAP [41, 42, 43]. This could be due to lower baseline level in the pre‐operative state of participants. This was supported by Gailey et al. who stated that the removal of limiting factors such as socket related pain and discomfort could lead to overestimation of results [34].

Similarly, studies assessing Patient‐Reported Outcome Measures (PROMs), such as PLUS‐M and ABC, found no significant differences between the BAP group and the SSP group [29, 34, 35]. This suggests that both types of prostheses offer comparable balance confidence, reaching levels similar to those of able‐bodied individuals (*p* = 0.095) [35].

Interestingly, the PMQ2.0 score in this review was significantly higher in the BAP group compared to the SSP group [33, 36]. A possible explanation for this could be due to the retrospective nature of data collection, along with the fact that both studies were conducted by the same author, potentially introducing selection bias.

### 4.2 Quality of Life

Most of the included studies reported no significant difference in Q‐TFA [29, 31, 35] and SF‐36 scores [31]. This suggests that SSP users were highly functional and satisfied with their prosthetic treatment [31].

Notably, Pospiech et al. reported a significantly lower Problem score for BAP users [37]. This could be attributed to the fact that the Q‐TFA score was originally designed for SSP users, focusing on common socket‐related problems. However, the Q‐TFA does not account for postoperative infections, which is a more relevant concern for BAP users, potentially overestimating the results [37]. This limitation is reflected in the meta‐analysis, which showed substantial heterogeneity (*I* ^2^ = 57%), likely reflecting the limited applicability of the Q‐TFA in capturing BAP‐specific issues.

Furthermore, Pospiech et al. reported conflicting results between the Q‐TFA and EQ‐5D‐3L. While general QoL, as measured by the EQ‐5D‐3L, showed no significant difference between groups, prosthesis‐related QoL, assessed using the Q‐TFA Global score, was significantly higher in the BAP group [37]. These findings suggest that while BAP may enhance prosthesis satisfaction, as measured with the Q‐TFA, it does not necessarily result in a significant improvement in overall QoL [37].

### 4.3 Gait

Overall, no significant differences were identified for gait parameters such as single‐support time, step length, step time, and gait speed [30, 31, 32, 34]. This suggests that SSP have a limited impact on gait mechanics and the benefits of BAP observed in individuals with socket‐related issues cannot be generalized to all SSP users [30]. This is further supported by Gailey et al., who suggest that BAP should be an alternative for amputees who have limitations with SSP [34].

In contrast, only both Welke et al. and Ranaldi et al. reported a significantly lower step width in the BAP group [31, 32]. This reduction in step width may enhance gait stability, promote a more symmetrical gait pattern, and reduce joint overuse in the hip and knee [31, 32]. As supported by other biomechanical studies, improved symmetry and coordination have been associated with more balanced load distribution across limbs, thereby reducing compensatory loading and joint overuse in the hip and knee and lowering the risk of secondary musculoskeletal complications [44, 45, 46, 47].

### 4.4 Hip ROM

Based on the results of two studies, the BAP group showed consistently higher ROM compared to the SSP group, though it is inconclusive on whether the difference is significant [31, 40]. A likely reason for the increase in ROM could be due to different muscle activation in SSP and BAP users. Pantall et al. showed that SSP users continuously engage muscles for stability, causing fatigue, while BAP users use muscles solely for walking, enabling more efficient movement [48].

### 4.5 Osseoperception

Haggstrom et al. reported that the BAP group detected more frequency levels than the SSP group, with significant improvements in higher frequencies for BAP. However, the clinical significance of these frequency levels remains unclear. It is suggested that increased sensory feedback through the prosthesis may help patients detect its position, potentially improving balance and reducing the risk of falls [38].

### 4.6 Cost Analysis

This review reported that the annual mean cost of BAP was not significantly lower compared to SSP. This modest cost difference may be due to the higher expense of BAP components, which provide more stable anchorage and potentially offer greater long‐term benefits for patients [39].

This is supported by Frossard et al., who conducted a cost‐modeling study using simulated data and suggested that, while BAP may have higher costs, their potential to improve mobility and comfort could make them a worthwhile investment, particularly for individuals with higher mobility levels [49]. Other studies similarly indicate that higher upfront costs may be offset by functional improvements and potential long‐term economic benefits [50, 51]. This is consistent with the literature, suggesting that BAP is primarily intended for patients with socket‐related issues and have good bone health [5].

However, it is important to recognize that comparing the cost of prosthetic services is complex due to variations in healthcare systems worldwide [39]. Hence, further research is needed to explore whether the higher initial costs of BAPs could be offset by potential gains in quality‐adjusted life‐years (QALYs) [49].

## 5 Limitations

This review has several limitations. Since only cross‐sectional studies were included, there is a lack of longitudinal data to assess changes in outcomes over time or the adaptation process to prostheses. Additionally, there is considerable variation in the duration participants had used their prostheses, with most studies reporting under 10 years' usage. This timeframe may not be sufficient to capture the long‐term benefits and potential complications associated with BAP. Future studies with extended follow‐up periods are necessary to provide more comprehensive insights into long‐term outcomes.

Selection bias is another limitation of this review. The strict eligibility criteria for BAP surgery mean that BAP users may not be fully representative of the broader amputee population. Most studies included in this review involved predominantly individuals with traumatic amputations, which likely reflects the patient selection criteria for BAP implantation. Individuals with vascular disease, poor bone quality, or significant comorbidities are often excluded from BAP surgery. As outcomes following amputation differ substantially by underlying etiology, the findings of this review should not be generalized to all individuals with amputation. Pooling individuals with different causes of amputation may obscure clinically relevant differences in mortality, complication rates, and long‐term outcomes. Future studies should therefore perform subgroup analyses based on the underlying cause of amputation, particularly comparing traumatic and disease‐related amputations, to better characterize potential differences in outcomes.

Furthermore, several important confounding variables may have influenced the findings of this review. In particular, key factors such as prosthetic knee type and patient functional level were not consistently reported, despite their influence on mobility and functional outcomes. Differences in rehabilitation protocols, patient demographics, and implant design (e.g., screw‐type versus press‐fit systems) may also have contributed to heterogeneity in results.

The lack of consistent reporting of these variables limited the ability to perform stratified analyses. There is also limited number of studies reporting on each outcome measure reduces the reliability of the results. Consequently, the findings should be interpreted with caution. Future studies should incorporate these variables and perform appropriate stratified analyses to better determine their impact on clinical and functional outcomes.

## 6 Conclusion

In cross‐sectional comparisons, BAP do not outperform SSP for mobility, gait speed, or global quality of life. Possible advantages in hip ROM and osseoperception are small and inconsistently measured. Future research should use large, prospective cohort studies with standardised outcome measures, include comparative complications (including infection), and incorporate cost‐utility analyses to define which patients derive the greatest net benefit.

## Author Contributions

All authors had full access to the data in the study and took responsibility for the integrity of the data and the accuracy of the data analysis. Conceptualization: Janice Tan Sue Wei. Methodology: Janice Tan Sue Wei and Wen Xian Low. Investigation: Janice Tan Sue Wei and Wen Xian Low. Formal analysis: Janice Tan Sue Wei and Wen Xian Low. Resources: Janice Tan Sue Wei. Writing – original draft: Janice Tan Sue Wei. Writing – review and editing: Janice Tan Sue Wei. Wen Xian: Chris Bretherton. Visualization: Janice Tan Sue Wei and Chris Bretherton. Supervision: Chris Bretherton. Funding acquisition. NIHR Barts Biomedical Research Centre.

## Funding

All authors take responsibility for the integrity of the data and the accuracy of the analysis. This research was supported by the NIHR Barts Biomedical Research Centre (NIHR203330).

## Disclosure

All authors have met the authorship criteria according to the latest guidelines of the International Committee of Medical Journal Editors, and all authors are in agreement with the manuscript.

## Consent

The authors have nothing to report.

## Conflicts of Interest

The authors declare no conflicts of interest.

## Supporting information


**Appendix S1:** Assessment of level of evidence with GRADEpro tool.

**Appendix S2:** os70369‐sup‐0002‐AppendixS2.xlsx.

## Data Availability

The data that supports the findings of this study are available in the Supporting Information S1 and S2 of this article.
