# Supplementary material for: Clinical Outcomes of Bone‐Anchored Versus Socket‐Suspended Prostheses in Individuals With Transfemoral Amputation: A Systematic Review and Meta‐Analysis of Two‐Arm Comparative Studies
Source: Orthop Surg. 2026 Jul 2;18(8):1545–62. doi: 10.1111/os.70369 (PMC13399259; doi:10.1111/os.70369)
Supplement: Supplementary file 1 — Appendix S1: Assessment of level of evidence with GRADEpro tool. [file OS-18-1545-s001.docx]

| **Certainty assessment** | | | | | | | **№ of patients** | | **Effect** | | **Certainty** | **Importance** |
| --- | --- | --- | --- | --- | --- | --- | --- | --- | --- | --- | --- | --- |
| **№ of studies** | **Study design** | **Risk of bias** | **Inconsistency** | **Indirectness** | **Imprecision** | **Other considerations** | **BAP** | **socket prostheses** | **Relative (95% CI)** | **Absolute (95% CI)** |  |  |
| **Mobility (assessed with: TUG)** | | | | | | | | | | | | |
| 3 | non-randomised studies | serious^a,b,c^ | not serious | not serious | serious^d^ | none | 41 | 38 | - | MD **0.16 higher** (2.09 lower to 2.41 higher) | ⨁◯◯◯ Very low^a,b,c,d^ | IMPORTANT |
| **Mobility (assessed with: ABC)** | | | | | | | | | | | | |
| 3 | non-randomised studies | serious^a,b,c^ | not serious | not serious | serious^d^ | none | 29 | 29 | - | MD **0.05 higher** (22.19 lower to 22.29 higher) | ⨁◯◯◯ Very low^a,b,c,d^ | IMPORTANT |
| **Moblilty (assessed with: 6MWT)** | | | | | | | | | | | | |
| 2 | non-randomised studies | serious^a,b,c^ | not serious | not serious | serious^d^ | none | 28 | 25 | - | mean **65 lower** (0 to 0 ) | ⨁◯◯◯ Very low^a,b,c,d^ | IMPORTANT |
| **Mobility (assessed with: 2MWT)** | | | | | | | | | | | | |
| 1 | non-randomised studies | serious^a,b,c^ | not serious | not serious | serious^e^ | none | 10 | 10 | - | mean **7 lower** (0 to 0 ) | ⨁◯◯◯ Very low^a,b,c,e^ | IMPORTANT |
| **Mobility (assessed with: PLUS-M)** | | | | | | | | | | | | |
| 2 | non-randomised studies | serious^a,b,c^ | not serious | not serious | serious^d,f^ | none | - | - | - | mean **0.34 higher** (0 to 0 ) | ⨁◯◯◯ Very low^a,b,c,d,f^ | IMPORTANT |
| **Mobility (assessed with: PMQ2.0)** | | | | | | | | | | | | |
| 2 | non-randomised studies | serious^a,b,c^ | not serious | not serious | serious^d,f^ | publication bias strongly suspected^g^ | Both studies showed significant results with the BAP group compared to the socket group, but raw data not available for one of the studies | | | | ⨁◯◯◯ Very low^a,b,c,d,f,g^ | IMPORTANT |
| **Quality of Life (assessed with: Q-TFA global)** | | | | | | | | | | | | |
| 3 | non-randomised studies | serious^a,b,c^ | serious^h^ | not serious | serious^d,i^ | none | 52 | 44 | - | MD **1.14 higher** (22.02 lower to 24.3 higher) | ⨁◯◯◯ Very low^a,b,c,d,h,i^ | IMPORTANT |
| **Quality of Life (assessed with: Q-TFA Prosthetic Mobility)** | | | | | | | | | | | | |
| 3 | non-randomised studies | serious^a,b,c^ | not serious | not serious | serious^d,i^ | none | 52 | 44 | - | MD **0.37 higher** (9.93 lower to 10.68 higher) | ⨁◯◯◯ Very low^a,b,c,d,i^ | IMPORTANT |
| **Quality of Life (assessed with: Q-TFA Problem)** | | | | | | | | | | | | |
| 3 | non-randomised studies | serious^a,b,c^ | serious^h^ | not serious | serious^d,i^ | none | 52 | 44 | - | MD **6.29 lower** (18.77 lower to 6.19 higher) | ⨁◯◯◯ Very low^a,b,c,d,h,i^ | IMPORTANT |
| **Quality of Life (assessed with: Q-TFA Prosthetic use)** | | | | | | | | | | | | |
| 4 | non-randomised studies | serious^a,b,c^ | not serious | not serious | serious^i^ | none | 60 | 52 | - | MD **1.18 higher** (2.36 lower to 4.71 higher) | ⨁◯◯◯ Very low^a,b,c,i^ | IMPORTANT |
| **Quality of Life (assessed with: EQ-5D)** | | | | | | | | | | | | |
| 2 | non-randomised studies | serious^a,b,c^ | serious^j^ | not serious | serious^d^ | none | Pospiech et al. reported no significant differences in EQ-5D scores for TTO-VS (*p* = 0.723) or VAS-VS (*p* = 0.497). Orgel et al. reported a significant improvement in the BAP group (*p* = 0.004). | | | | ⨁◯◯◯ Very low^a,b,c,d,j^ | IMPORTANT |
| **Quality of Life (assessed with: SF-36)** | | | | | | | | | | | | |
| 1 | non-randomised studies | serious^b,c^ | serious^k^ | not serious | serious^e^ | none | The study showed no significant difference at both the PCS (*p* = 0.892) and MCS (*p* = 0.293) score | | | | ⨁◯◯◯ Very low^b,c,e,k^ | IMPORTANT |
| **Quality of Life (assessed with: SAT-PRO)** | | | | | | | | | | | | |
| 1 | non-randomised studies | serious^c^ | serious^k^ | not serious | serious^e^ | none | The study showed a significant improvement within the BAP group (*p* = 0.000). | | | | ⨁◯◯◯ Very low^c,e,k^ | IMPORTANT |
| **Gait (assessed with: Walking speed)** | | | | | | | | | | | | |
| 3 | non-randomised studies | serious^a,b,c^ | not serious | not serious | serious^d^ | none | 39 | 38 | - | MD **0.03 lower** (0.31 lower to 0.25 higher) | ⨁◯◯◯ Very low^a,b,c,d^ | IMPORTANT |
| **Gait (assessed with: Single Support)** | | | | | | | | | | | | |
| 2 | non-randomised studies | serious^b,c^ | not serious | not serious | serious^d^ | none | Both studies reported no significant difference at (*p* = 0.387) and (*p* = 0.95) respectively. | | | | ⨁◯◯◯ Very low^b,c,d^ | IMPORTANT |
| **Gait (assessed with: Step Length)** | | | | | | | | | | | | |
| 3 | non-randomised studies | serious^b,c^ | not serious | not serious | serious^d^ | none | Two studies reported no significant difference at (*p* = 0.693) and (*p* = 0.574) respectively. The other one reported no *p-* value but stated there was no significant difference. | | | | ⨁◯◯◯ Very low^b,c,d^ | IMPORTANT |
| **Gait (assessed with: Step Time)** | | | | | | | | | | | | |
| 2 | non-randomised studies | serious^b,c^ | not serious | not serious | serious^d^ | none | One study reported no significant difference at (*p* = 0.882). The other one reported no *p-* value but stated there was no significant difference. | | | | ⨁◯◯◯ Very low^b,c,d^ | IMPORTANT |
| **Gait (assessed with: Step width)** | | | | | | | | | | | | |
| 3 | non-randomised studies | serious^b,c^ | serious^l^ | not serious | serious^d^ | none | Two studies reported significant difference at (*p* = 0.001) respectively. The other one reported no *p-* value but stated there was no significant difference. | | | | ⨁◯◯◯ Very low^b,c,d,l^ | IMPORTANT |
| **Hip Range of Motion (assessed with: Flexion-extension)** | | | | | | | | | | | | |
| 2 | non-randomised studies | serious^a,b,c^ | serious^m^ | not serious | serious^d^ | none | 40 | 60 | - | MD **16.95 higher** (0 to 0 ) | ⨁◯◯◯ Very low^a,b,c,d,m^ | IMPORTANT |
| **Hip Range of Motion (assessed with: Abduction-adduction)** | | | | | | | | | | | | |
| 2 | non-randomised studies | serious^a,b,c^ | not serious | not serious | serious^d^ | none | 40 | 60 | - | MD **4.1 higher** (0 to 0 ) | ⨁◯◯◯ Very low^a,b,c,d^ | IMPORTANT |
| **Hip Range of Motion (assessed with: Rotation)** | | | | | | | | | | | | |
| 2 | non-randomised studies | serious^a,b,c^ | serious^m^ | not serious | serious^d^ | none | 40 | 60 | - | MD **24.3 higher** (0 to 0 ) | ⨁◯◯◯ Very low^a,b,c,d,m^ | IMPORTANT |
| **Osseoperception** | | | | | | | | | | | | |
| 2 | non-randomised studies | serious^a,b,c^ | serious^j,m^ | not serious | serious^d^ | none | One study showed that the BAP group had better ability to detect high frequency vibrations (125 and 250 Hz) than the socket group (*p* = 0.01 and 0.03, respectively). There was no significant different between groups at the lower frequencies. Another study showed BAP group scored significantly higher across V1–V3 (7.1 ± 1.1) compared to the Socket group (5.4 ± 1.2, *p* < 0.001). | | | | ⨁◯◯◯ Very low^a,b,c,d,j,m^ | IMPORTANT |
| **Cost** | | | | | | | | | | | | |
| 1 | non-randomised studies | extremely serious^b,c,m,n^ | serious^k^ | not serious | serious^e^ | none | The BAP group had 14% lower mean annual costs for new prostheses, services, repairs, and adjustments (p = 0.632). The socket group also had significantly more annual visits compared to the BAP group (*p* < 0.0001). The socket group had significantly more annual visits compared to the BAP group (*p* < 0.0001). | | | | ⨁◯◯◯ Very low^b,c,e,k,m,n^ | IMPORTANT |

**CI:** confidence interval; **MD:** mean difference

#### Explanations

a. Bias due to confounding.

b. Bias in classification of intervention

c. Bias in measurement of outcome

d. Less than three studies included giving small sample size

e. Only one study reported so small sample size

f. No raw data provided for one of the studies

g. Same author for both studies

h. High heterogeneity (I² > 50%) in forest plot

i. One study not included in meta-analysis as no raw data provided but reported significant result

j. Different outcome measurement are used giving variation in results

k. No variation between studies as only one study included

l. Two studies reported significant difference, the other one reported no significant difference

m. Variation in results. One study reported significant difference, the other reported no significant difference

n. Bias in selection of reported result
